# Multi-Targeting Approach in Glioblastoma Using Computer-Assisted Drug Discovery Tools to Overcome the Blood–Brain Barrier and Target EGFR/PI3Kp110β Signaling

**DOI:** 10.3390/cancers14143506

**Published:** 2022-07-19

**Authors:** Catarina Franco, Samina Kausar, Margarida F. B. Silva, Rita C. Guedes, Andre O. Falcao, Maria Alexandra Brito

**Affiliations:** 1 LASIGE, Department of Informatics, Faculty of Sciences, Universidade de Lisboa, Campo Grande, 1749-016 Lisboa, Portugal; ca.franco@campus.fct.unl.pt (C.F.); saminakausar.bioinfo@gmail.com (S.K.); 2 Research Institute for Medicines, Faculty of Pharmacy, Universidade de Lisboa, Av. Prof. Gama Pinto, 1649-003 Lisboa, Portugal; mbsilva@ff.ulisboa.pt (M.F.B.S.); rguedes@ff.ulisboa.pt (R.C.G.); 3 Department of Pharmaceutical Sciences and Medicines, Faculty of Pharmacy, Universidade de Lisboa, Av. Prof. Gama Pinto, 1649-003 Lisboa, Portugal

**Keywords:** blood–brain barrier, dual-targeting, epidermal growth factor receptor, glioblastoma, phosphatidylinositol-3-kinase, quantitative structure–activity relationship models, virtual screening

## Abstract

**Simple Summary:**

Treatment of glioblastoma is hampered by the activation of compensatory survival mechanisms by malignant cells that lead to drug resistance. Moreover, the blood–brain barrier (BBB) precludes the brain entrance of most drugs. We hypothesized that computer-assisted drug discovery tools would reveal novel multi-targeting drug candidates with BBB-permeant and favorable ADMET properties. We aimed to discover molecules with predicted ability to inhibit the EGFR/PI3Kp110β pathway and to validate their efficacy and safety in biological assays. We used quantitative structure–activity relationship models and structure-based virtual screening, and assessed ADMET properties, to identify BBB-permeant drug candidates. Moreover, we tested their anti-tumor efficacy and BBB safety and permeation in cell models. We found two EGFR, two PI3Kp110β, and, mostly, two dual inhibitors with anti-tumor effects. Among them, one EGFR and two PI3Kp110β inhibitors were able to cross the BBB endothelium without compromising it. These studies revealed novel drug candidates for glioblastoma treatment.

**Abstract:**

The epidermal growth factor receptor (EGFR) is upregulated in glioblastoma, becoming an attractive therapeutic target. However, activation of compensatory pathways generates inputs to downstream PI3Kp110β signaling, leading to anti-EGFR therapeutic resistance. Moreover, the blood–brain barrier (BBB) limits drugs’ brain penetration. We aimed to discover EGFR/PI3Kp110β pathway inhibitors for a multi-targeting approach, with favorable ADMET and BBB-permeant properties. We used quantitative structure–activity relationship models and structure-based virtual screening, and assessed ADMET properties, to identify BBB-permeant drug candidates. Predictions were validated in in vitro models of the human BBB and BBB-glioma co-cultures. The results disclosed 27 molecules (18 EGFR, 6 PI3Kp110β, and 3 dual inhibitors) for biological validation, performed in two glioblastoma cell lines (U87MG and U87MG overexpressing EGFR). Six molecules (two EGFR, two PI3Kp110β, and two dual inhibitors) decreased cell viability by 40–99%, with the greatest effect observed for the dual inhibitors. The glioma cytotoxicity was confirmed by analysis of targets’ downregulation and increased apoptosis (15–85%). Safety to BBB endothelial cells was confirmed for three of those molecules (one EGFR and two PI3Kp110β inhibitors). These molecules crossed the endothelial monolayer in the BBB in vitro model and in the BBB-glioblastoma co-culture system. These results revealed novel drug candidates for glioblastoma treatment.

## 1. Introduction

Estimates indicate that around 77% of primary brain tumors are gliomas and that glioblastoma (GB) is the most malignant and frequent type of glioma [1], with more than 10,000 cases annually diagnosed and a 95% 5-year mortality [2]. To this poor prognosis accounts GB pathophysiology heterogeneity, treatment refractoriness, and a high rate of recurrence due to the extensive invasiveness of the surrounding cerebral parenchyma, which renders it difficult to complete surgical resections [3].

Within a broad pool of redundant pathways and potential targets [4], amplification and mutation of the epidermal growth factor receptor (*EGFR* or *HER1*) gene arise in about 60% of primary GB, thus becoming an attractive therapeutic target [5]. EGFR overexpression has been associated with decontrolled cell proliferation and inhibition of apoptosis, as well as with the establishment of malignancy by inducing the progression of low-grade to high-grade glioma [6]. Thus, a search for new EGFR tyrosine kinase inhibitors (TKIs) has been pursued [7], but monotherapy with these agents has not increased disease-free survival [8]. Several studies have pointed out that activation of compensatory pathways that generate multiple inputs to downstream phosphatidylinositol-3-kinase (PI3K) signaling may confer insensitivity to EGFR-targeted therapies, unraveling new opportunities for multitargeting in malignant and resistant brain tumors [9]. Actually, the PI3K/AKT/mTOR pathway is overexpressed or constitutively hyperactivated in about 78% of GB cases, strongly contributing to tumor malignancy and invasiveness [10]. The least studied PI3Kp110β subunit was recently described as the principal activator of downstream AKT signaling with a strong association with tumors’ recurrence rate and prognosis [11]. However, there are no published successful results indicating the relevance of PI3Kp110β inhibition in glioma cells or proposing EGFR/PI3K dual inhibitors.

Most drugs showing pre-clinical potential against GB cannot achieve therapeutic concentrations at the tumor site due to low blood–brain barrier (BBB) penetration [12]. The anatomic basis of the BBB is formed by a tightly sealed monolayer of brain microvascular endothelial cells. These cells have unique features such as the expression of complex intercellular junctions that are responsible for high transendothelial electrical resistance (TEER) and low paracellular flux, which reflect a restricted permeability [13]. So, approximately 100% of large molecules and 98% of small molecules do not cross the BBB [13], dampening the treatment of most brain pathologies.

Only low-molecular-weight (<700 Da but typically <300 Da) lipophilic molecules may diffuse through BBB’s endothelium and enter the brain passively [14]. However, drugs with high lipid-solubility bind to plasma proteins such as albumin, with a low off-rate, which restricts their delivery to the brain [15]. Moreover, increased lipophilicity raises the likelihood of the drug becoming a substrate for BBB efflux transporters, such as the highly expressed P-glycoprotein (P-gp), restricting the drug’s accumulation in the brain [16]. These facts have attracted attention to study the BBB as an obstacle to overcome during the drug discovery phase, which could represent the key for the successful treatment of neuro-pathologies such as GB [17].

Aware that drug discovery is a challenging, time consuming, and expensive process, computational tools may act as a “virtual shortcut”, speeding up and potentially reducing the research cost. Through the use of specialized machine learning algorithms and access to large libraries of compounds, in silico testing allows the screening of a high number of molecules in search of those that fit optimal queries and cause the desired biologic response [18]. Computational modeling is able to support multitargeting approaches by predicting BBB permeation, target-specificity, and ADMET (absorption, distribution, metabolism, excretion, and toxicity) properties of molecules, opening doors to a new dimensionality of targeted and combined therapies, with higher effectiveness, for many brain pathologies, namely GB [19].

Considering the continuous amount of molecular data being added to compound activity databases such as ChEMBL or PubChem, there are emerging opportunities to build well-trained and properly validated empirical models [20]. A common molecular representation used in these models relies on the use of chemical fingerprints that generally identify molecular fragments such as molecular descriptors encoded as bit vectors, where each bit position is associated with a presence or absence of a specific substructure pattern [21]. Several supervised machine learning tools are able to use this type of representation for pattern recognition algorithms, allowing to build automated and robust Quantitative Structure–Activity Relationship (QSAR) models by correlation of molecular structural information with biological activities [22]. Such models have limitations from the limited data from which they were fitted. So, the concept of an applicability domain (AD) defines the structural boundaries to which a model can be reliably applied [23]. Moreover, there is a trend to improve the quality of in silico predictions by allying the empirical results of QSAR models and similarity searches to 3D-docking simulations, providing a direct target–ligand interaction study to unravel the energetically most favorable binding mode [24,25]. The combination of computational approaches with experimental practices and clinical trials has revealed strategic primacies to overcome medicinal chemistry challenges and assess the efficacy and safety of new medical interventions, minimizing the ethical concerns, and boosting pharmaceutical research productivity and quality [20,26].

The aim of this work is to identify drug candidates that predictably overcome the BBB and inhibit the EGFR/PI3Kp110β pathway as a multi-targeting approach against GB. We used an in silico strategy, relying on automated QSAR models and docking simulations, to screen libraries of millions of molecules, as well as using relevant in vitro models and biological assays to validate the obtained computational results. We succeeded in identifying promising drug candidates, in this way opening new avenues for GB treatment.

## 2. Materials and Methods

### 2.1. In Silico Strategy for Hits Identification

#### 2.1.1. Automated Framework for QSAR Model Building

**Figure 1:**
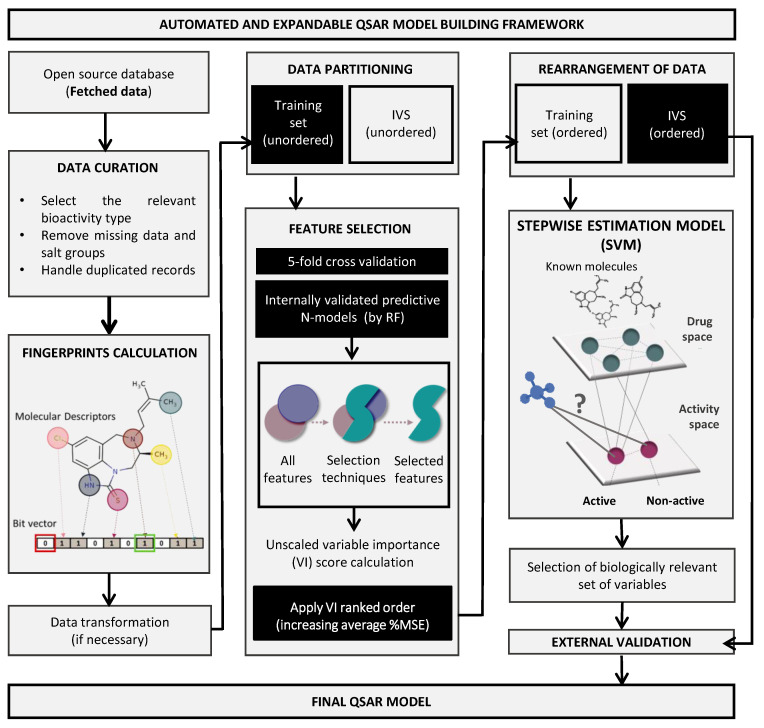
QSAR model building pipeline. KNIME automated framework for model building embeds all tools necessary to perform the entirely QSAR life cycle. The workflow was built to automatically access and process fetched molecular data for a given target or problem, calculate descriptors and fingerprints, select optimized set of features by using an enhanced random forest (RF) methodology, and follow an unbiased protocol for QSAR models’ internal and external validation using reliable machine learning algorithms, as the support vector machine (SVM). IVS, independent validation set; MSE, mean square error.

An automated and expandable framework for QSAR models’ building was used based on our previous work [20]. The open-source software KoNstanz Information MinEr (KNIME) was selected due to its graphical interface with several customized nodes [27]. R (version 3.4.4) [28] was the open-source programming language chosen to perform the statistical evaluations. An overview of the used framework is shown in Figure 1. The complete process was divided into several systematic tasks of QSAR modeling, covered in the following sections.

##### Training Data Access and Processing

For BBB permeation model building, a dataset with experimental records about the logBB (logarithmic ratio between the concentration of a drug in brain and blood sides) was used [29]. For EGFR and PI3Kp110β inhibition models, IC_50_ data were obtained from ChEMBL [30]. Data curation included filtering out missing data, handling of duplicates, and exclusion of salt groups. The relevant information about the used datasets is summarized in Supplementary Table S1.

##### Data Transformation and Partitioning

For coding molecular descriptors, the RDKit node [31] was used for vector representations by computing fingerprints. Atom Pair Fingerprints were selected due to better performance than Morgan Fingerprints [32].

To standardize highly varying values in raw data for proper training of predictive models, IC_50_ values were scaled (spIC_50_) by logarithmic transformation (1). This transformation was skipped for the BBB permeation dataset since the data were already normalized (logBB).

(1) $$\{\begin{array}{c} {\mathrm{IC}}_{50}\geq 10,000\;\mathrm{nM},\;{\mathrm{spIC}}_{50}=0\\ 1\;\leq {\mathrm{IC}}_{50}\leq 10,000\;\mathrm{nM},\;{\mathrm{spIC}}_{50}=\frac{4-\log_{10}({\mathrm{IC}}_{50})}{4}\\ \;{\mathrm{IC}}_{50}\leq 1.0\;\mathrm{nM},\;{\mathrm{spIC}}_{50}=1.0 \end{array}$$

For analysis purposes, the values were converted in -pIC_50_ scale (1 to 10) (2):

−pIC_50_ = −log_10_(1 × 10^−9^ × (10^4−4sp(IC50)^)) (2)

The processed data were divided into 75% training set and 25% independent validation set (IVS) through a random sampling. The training set was further used for feature selection and internal model evaluation, while the IVS was used for external validation after the best model for each chemical problem was built and selected [33].

##### Feature Selection

A hybrid approach was used for feature selection entailing a random forest (RF) voting procedure to rank variables according to their importance [20,34]. The unscaled variable importance (VI) score was counted considering the mean squared error (MSE) averaged from each decision tree with random sampling and replacement of given variables.

##### Model Building

The VI-based ordered training data were further processed to be fed to the non-linear supervised learning support vector machine (SVM) algorithm and to build stepwise fitted N-regression QSAR models. The internal validation of the results was established by 5-fold cross-validation. R package e1071 was used for SVM regression using the default parameters. The ranked variables were sequentially added to the learning algorithm, and the best features based internally validated model was selected for external validation. Parallelly, the same QSAR modeling workflow fitted a model with the whole set of descriptors.

##### External Validation and Models’ AD

After selecting the predictive model with the best set of selected features (SF-model), unbiased external validation was performed using IVS. The predictive power was assessed by comparing the proportion of the variance explained (PVE), and the root mean squared error (RMSE) ((3) and (4)),

(3) $$\mathrm{PVE}=1-\frac{\sum_{i=1\;}^{n}{\left(\mathrm{yi}\;-\;ýi\right)}^{2}}{\sum_{i=1\;}^{n}{\left(\mathrm{yi}\;-\;ÿi\right)}^{2}}$$

(4) $$\mathrm{RMSE}=\sqrt{\frac{1}{N}\sum_{i=1\;}^{n}{\left(\mathrm{yi}-ýi\right)}^{2}}$$

where yi and ýi are the measured and predicted biologically associated values for compound i, respectively, and ÿ is the mean of all compounds’ activities in the dataset.

A KNIME “Domain-Similarity” node was used to measure molecular distances between the IVS and the training set. The prediction was considered unreliable if the distance of an IVS molecule to its nearest neighbor in the training set was higher than the similarity threshold of 0.7. The externally validated final models were then used as a tool for external prediction and virtual screening.

#### 2.1.2. ZINC15 Virtual Screening

A purchasable subset of molecules from ZINC15 [35] was used for virtual screening locally. Data were processed as described above. The ZINC15 screening output was filtered out by a PAINS (pan-assay interference compounds) filter to decrease the number of false positives [36]. For each target, the best-scored molecules were plotted by predicted molecular activity vs. BBB calculated permeation, and the most promising ones were selected.

#### 2.1.3. Molecular Docking and Scoring

To analyze the interaction between selected molecules and respective targets, several docking protocols were tested, as briefly covered in following sections.

##### Data Access and Processing

The structural data about the catalytic domains of EGFR (P00533) and PI3Kp110β (P42338) were obtained from UniProtKB. Several X-ray 3-D structures for EGFR (PDBID: 1xkk, 3w2s, 3poz, and 5u8l) and PI3Kp110β (PDBID: 2y3a and 4bfr) were selected from Protein Data Bank (PDB) and imported into Molecular Operating Environment (MOE2018). Relevant information about such PDB’s is summarized in Supplementary Table S2. The 3D structures were protonated, aligned, and superposed for the inspection of catalytic pockets.

##### Docking Protocol Selection and Validation

Two different softwares, MOE and Genetic Optimization for Ligand Docking (GOLD), were tested in self- and cross-docking studies. In MOE, docking was run with the following parameters: placement (triangle matcher), refinement (rigid receptor), and poses (500). “Free energies” of binding were obtained by the following score functions: GBVI/WSA dG, Alpha HB, and London dG. In GOLD, a full range of ligand flexibility with partial protein flexibility was explored in an automated way using a stochastic method. To address binding affinities the following score functions were tested: GoldScore, ChemScore, ASP, and ChemPLP. The GOLD docking protocol considered 1000 poses, and the center of the docking calculations was fixed by the nitrogen atom both at Met793 in EGFR structures, and Lys799 in PI3Kp110β structures.

The docking protocol for each protein system was chosen according to the best alignment and superposition between docked and crystallographic ligands, translated by the lowest root mean square deviation (RMSD), and the ability to distinguish between active and inactive molecules. RMSD calculations were performed using PyMOL software, where the 3D representative images were also constructed. For docking protocols validation, standard curves were built (IC_50_ vs. score). Control molecules were washed, protonated and energy minimized using default parameters. The results were analyzed by linear regression, after which ZINC15 screened molecules were docked in final 3D models according to unbiased protocol.

#### 2.1.4. ADMET Properties Analysis

Maestro software (Schrödinger, Release 2015–4, LLC, New York, NY, USA, 2014) was used to create 3D models of candidate molecules, cutoff as an input for QikProp application to estimate several theoretical descriptors relevant for molecules’ BBB transport, and a drug-like phenotype. Some ADMET parameters were also predicted using the open-source AdmetSAR2.0.

#### 2.1.5. Molecule’s Selection

The selection of the screened molecules that moved into the pre-clinical assays was performed according to the following criteria: QSAR models’ predictions of EGFR/PI3Kp110β activity and BBB’s permeation; score from docking simulation; ADMET properties. Conversely, the following criteria were used to exclude molecules: not available for purchase in MolPort Inc. supplier; already published in ChEMBL database as inhibitors of considered targets or similar; classified as being out of models’ AD; classified as PAINS.

### 2.2. Cell-Based Methods for In Silico Strategy Validation and Lead Compounds Identification

#### 2.2.1. Cell Lines and Conditions

A cell line of human brain microvascular endothelial cells (HBMEC) was used as a simplified model of the human BBB, as usual in our lab [37]. Two GB cell lines were used as GB models, namely U87MG parental and U87MG transfected to overexpress wild-type EGFR (U87MG-wtEGFR), kindly donated by Dr Vasco Branco, Research Institute for Medicines, Faculty of Pharmacy, Universidade de Lisboa, and Dr. Frank Furnari, Ludwig Institute for Cancer Research, San Diego, CA, USA, respectively. Both cell lines were grown in Dulbecco’s modified Eagle’s medium (DMEM), high glucose (Gibco, Waltham, MA, USA, Life Technologies, Carlsbad, CA, USA) supplemented with 10% fetal bovine serum (FBS, Biochrom AG, Berlin, Germany), and 1% antibiotic–antimycotic solution (Sigma Aldrich, Burlington, MA, USA), at 37 °C in a humidified atmosphere with 5% CO_2_.

#### 2.2.2. Molecule’s Preparation

All molecules were obtained from MolPort and dissolved in dimethyl sulfoxide (DMSO) at a stock concentration of 100 mM, aliquoted, and frozen at −20 °C.

#### 2.2.3. Screening Analysis of Cell Viability

To analyze molecules’ cytotoxicity, U87MG and U87MG-wtEGFR cells were seeded into 96-well plates at a density of 2.5 × 10^4^ cells/mL, and after 48 h were incubated with 0.1, 1, 10, or 100 µM of each molecule diluted in (non-supplemented) cell medium for 24 h. The half-maximal effective concentration (EC_50_) of the most cytotoxic compounds was quantified. Dose–response data were also obtained for treatment with triton X-100 (positive control) and DMSO (vehicle; negative control). The cytotoxicity of selected molecules from initial screening was tested in HBMEC at 100 µM for 24 h. HBMEC were seeded at a density of 8 × 10^4^ cell/mL in 96-well plates and after 48 h were incubated with the molecules.

In screening assays, cell viability was evaluated using thiazolyl blue tetrazolium bromide (MTT). Briefly, a culture medium containing 0.5 mg/mL of MTT was added to each well for 90 min at 37 °C. The supernatant was removed and the formed formazan crystals in viable cells were solubilized with 0.04 N HCl in isopropanol solution. The absorbance was recorded using a microplate reader (Zenyth 3100, Anthos Labtec Instruments, Salzburg, Austria) at 595 nm, and values of cell viability were calculated as percentage of negative control.

#### 2.2.4. Evaluation of Molecules’ BBB Transport

To evaluate molecules’ BBB transport, a well-validated two-chamber BBB model, relying on the use of a microporous semipermeable membrane (Transwell insert) that separates the upper (luminal: “blood side”) and the lower (abluminal: “brain side”) chambers, was used as usual in our team [37]. Briefly, HBMEC were seeded on polyester inserts (0.4 μm, Corning Costar Corp., New York, NY, USA) at a density of 8 × 10^4^ cell/insert and treated after 8 days in culture. Inserts were coated with rat-tail collagen-I (BD Biosciences, Erembodegem, Aalst, Belgium) before seeding. Transport assays were conducted in Hank’s Balanced Salt Solution (HBSS, Gibco, Waltham, MA, USA) with calcium and magnesium, supplemented with 0.1% FBS. Molecules were applied to the upper chamber at defined EC_50_ for 30 and 120 min, and transport analysis was performed as described below.

##### UPLC-MS/MS Analysis

Samples from upper and lower chambers were deproteinized using acetonitrile (ACN) (1:3) in ice-cold conditions, centrifuged at 15,300× *g* for 5 min at 4 °C and the respective supernatants were frozen until subsequent analysis.

The identification and quantification of molecules of interest were achieved by ultra-performance liquid chromatography–tandem mass spectrometry (UPLC-MS/MS) analysis using a triple quadrupole and Masslynx 4.1 software (Acquity TQ Waters). The separation of analytes was performed using a reversed-phase column Purospher STAR, RP-18 endcapped, 2 µm; Hibar HR 50-2.1 (Merck, Rahway, NJ, USA) at 35 °C, and a linear gradient (0.1% formic acid (A): ACN with 0.1% formic acid (B)) at a flow rate of 0.30 mL/min. The detection of analytes was based on electrospray ionization in the positive mode (ESI+) and acquisition in multiple reaction monitoring mode (MRM) at the following settings: ESI capillary voltage 3.0 kV, cone voltage 30 V, extractor voltage 1 V, RF lens voltage 0.1 V, desolvation gas flow 750 L/h at 350 °C, cone gas flow 50 L/h at 120 °C. Calibration curves were based on external standards using at least 8-points within the intended range, injected as duplicates. Analytes were quantified with a maximum deviation of 15% between the MRM1/MRM2 ratio. Each sample was injected in triplicate. Endothelial transport was calculated based on the ratio of lower compartment concentration and the sum of upper and lower compartments concentrations.

##### Evaluation of BBB Integrity in Transport Assays

HBMEC monolayer integrity was ensured during BBB transport assays by TEER measurements and sodium-fluorescein paracellular permeability assessment, as previously described [37]. TEER readings were performed using an EndOhm^TM^ chamber coupled to an EVOMX resistance meter (World Precision Instruments, Inc., Sarasota, FL, USA) and sodium fluorescein levels were measured using a Hitachi F-2000 fluorescence spectrophotometer.

#### 2.2.5. Measurement of Molecule’s Transport and Cytotoxicity in BBB-GB Model

To assess molecule cytotoxicity in GB cells after crossing the HBMEC monolayer, a co-culture BBB-GB model was used. HBMEC were seeded in the Transwell apparatus as described above. After 5 days, U87MG or U87MG-wtEGFR cells were seeded (1.2 × 10^5^ cell/mL) in the lower chamber. After 3 days of co-culture, upper chamber content was replaced with fresh medium (control) or medium containing each molecule at respective EC_50_. After 2 h, inserts were removed and tested for barrier integrity by TEER readings and immunofluorescence analysis, whereas GB cells were maintained in culture for 24 h to further assess cell viability by MTT assay.

#### 2.2.6. Assessment of Abluminal Conditioned Medium Effect in GB Cells

To evaluate disruptive effects in HBMEC monolayer when co-cultured, a transport assay using HBMEC monoculture was conducted. After 2 h of molecules incubation, the abluminal conditioned media were collected and used to supply U87MG or U87MG-wtEGFR cells for 24 h, which grew to confluence for 3 days. HBMEC were also tested for barrier integrity, and MTT assays were performed at the end of incubation.

#### 2.2.7. Immunofluorescence

To analyze target proteins expression, U87MG cells were seeded (1.2 × 10^5^ cell/mL) in coverslips, grown for 48 h, incubated with each molecule at EC_50_ for 1–9 h, and then stained for phospho-EGFR (1:100, Cell Signaling, Danvers, MA, USA, #2236), phospho-AKT (1:100, Thermo Fisher Scientific, Waltham, MA, USA, #700256), or both. To analyze the cytotoxic effect of molecules in HBMEC, the cells were seeded (8 × 10^4^ cell/mL) in rat-tail collagen-I-coated coverslips and after 48 h were treated with each molecule at EC_50_ for 9 h. HBMEC in coverslips were stained for: the junctional protein β-catenin (1:100, Thermo Fisher Scientific, Waltham, MA, USA, #71-2700), the cytoskeleton protein F-actin (1:1000, Phalloidin dye, Abcam, Cambridge, UK, #ab17675), and the efflux transporter P-gp (1:50, Santa Cruz Biotechnology, Dallas, TX, USA, #sc-390883). HBMEC seeded on polyester inserts for transport assays were also stained for the junctional protein zonula occludens-1 (ZO-1, 1:200, Thermo Fisher Scientific, Waltham, MA, USA, #40-2200) and the cytoskeleton protein vimentin (1:100, Thermo Fisher Scientific, Waltham, MA, USA, #MA3745).

Incubations with primary antibodies occurred overnight at 4 °C. Incubation with the secondary antibodies Alexa Fluor 555 (1:500, Thermo Fisher Scientific, Waltham, MA, USA, #A21428) or Alexa Fluor 488 (1:500, Thermo Fisher Scientific, Waltham, MA, USA, #A11001) lasted for 1 h at room temperature. Nuclei were counterstained with Hoechst 33342 dye (1:1000, Thermo Fisher Scientific, Waltham, MA, USA, #33342) for 10 min. Before mounting with DPX (Merck Millipore, Burlington, MA, USA), cells were dehydrated with methanol. Negative controls were performed.

#### 2.2.8. Microscopy and Image Analysis

Cell labeling was examined in an Olympus BX60 microscope, at the Faculty of Sciences, Universidade de Lisboa Microscopy Facility, a node of the Portuguese Platform for BioImaging (PPBI-POCI-01-0145-FEDER-022122). Morphological alterations were observed and documented under Hamamatsu’s analysis software. Fields were chosen at random.

All acquired images were treated in ImageJ (NIH, Bethesda, MD, USA). Assessment of nuclear morphology following Hoechst staining was evaluated [38]. For fluorescence intensity measures, the regions of interest were selected in Image J, and corresponding integrated densities were obtained. The representative 3D plots for BBB integrity were obtained by merged pixel intensity using the surface plot tool of Image J. For F-actin stress fibers analysis, the plot profile tool of Image J was used considering each defined cell-ferret diameter.

For analysis of β-catenin internalization and HBMEC morphological changes, Icy software was used. At least 5 cells per field were delineated and the conveyed values of membrane/interior pixels and roundness (area/perimeter^2^) were exported and analyzed. Taking advantage of membrane staining (β-catenin), the BBB’s integrity was also analyzed by quantifying the total area of intercellular gaps in the HBMEC monolayer.

### 2.3. Statistical Analysis

At least three independent experiments were performed and results are presented as means ± SEM. Significance of differences amongst treatments and controls were calculated by one-way ANOVA multiple comparison test with Tukey correction using GraphPad 8 software (GraphPad Software, San Diego, CA, USA). *p* < 0.05 was considered statistically significant.

## 3. Results

### 3.1. Internal and External Validation of Final QSAR Models Supported Their High Predictive Performance

**Figure 2:**
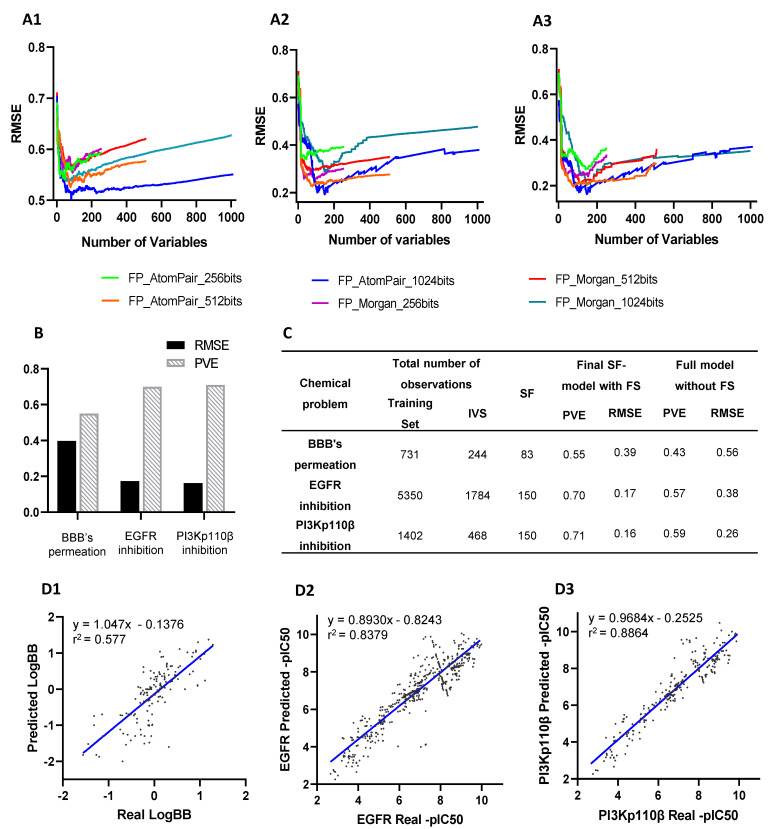
QSAR models building and validation: (**A**) The root mean square error (RMSE) delivered by models’ internal validation using each fingerprint (FP) type (Atompair and Morgan) and bit sizes (256, 512, or 1024) were compared between N-models developed by sequentially adding variables ranked according their importance score for each chemical problem, (**A1**) BBB’s permeation, (**A2**) EGFR inhibition, and (**A3**) PI3Kp110β inhibition. (**B**) Predictive performance assessment of externally validated final SF-models by respective RMSE and proportion of variance explained (PVE). (**C**) Comparison of the performance of externally validated final SF-models with full QSAR models (without feature selection (FS)) in predicting activity values of independent validation set (IVS). (**D**) Comparison of real values and predicted values of IVS by externally validated final SF-models for (**D1**) BBB’s permeation, (**D2**) EGFR inhibition, and (**D3**) PI3Kp110β inhibition. Correlation coefficients r^2^ were obtained by linear regression statistical analysis.

The performance of the presented QSAR modeling workflow was assessed by an unbiased protocol to support optimized predictive models for all chemical problems (Figure 2). The internal lowest RMSE in predicting BBB permeation (0.513), EGFR inhibition (0.193), and PI3Kp110β inhibition (0.175) was achieved by using AtomPair 1024-bit vectors, considering the 83 best-ranked features in BBB’s model and 150 features in the last two models (Figure 2A).

After selecting the best set of features and defining QSAR patterns, SF-models’ external validation was performed using an independent validation set (Figure 2B). The BBB permeation model was trained with a dataset of fewer than 1000 molecules, whereas EGFR and PI3K models were trained with 1870 and 7134 molecules (Supplementary Table S1), respectively.

The comparison between the performance of externally validated full-models (without feature selection) and final SF-models (Figure 2C) confirmed the effectiveness of feature selection, thus corroborating previous results [20]. The number of selected features in SF-models ranged between 8.1 and 14.6% of the total processed features considered in full models. The PVE score of full-models ranged between 0.43 and 0.59 while in final SF-models were in the 0.55–0.71 range. Moreover, error analysis of predictive models showed an averaged RMSE of full-models of 0.40, while the final SF-models had an averaged RMSE of 0.24.

A retroactive validation of the SF-model was accomplished using the IVS, showing an r^2^ coefficient of 0.577 in the BBB permeation model, 0.8387 in the EGFR model, and 0.8864 in the PI3Kp110β model (Figure 2D).

### 3.2. ZINC15 Screening by Developed QSAR Models Pointed out Unswerving Candidates

**Figure 3:**
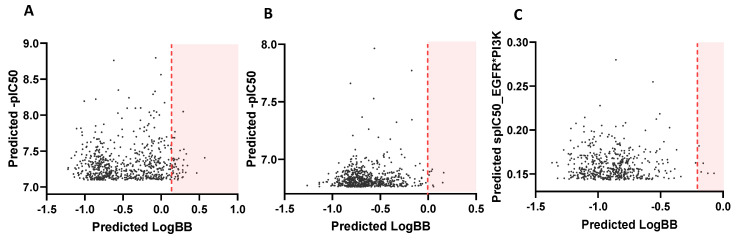
ZINC15 screening output. Each graphic was obtained by plotting the top thousand screened molecules for (**A**) EGFR inhibition, (**B**) PI3Kp110β inhibition, and (**C**) dual targeting. Orange lines mark the specified threshold for acceptable LogBB values and only hits from shaded area were selected.

The ZINC15 screening allowed the selection of: 31 molecules for EGFR with LogBB ≥ 0.2 and -pIC_50_ ≥ 7; 14 candidates for PI3Kp110β with LogBB ≥ 0 and -pIC_50_ ≥ 6.5; and 7 candidates for dual targeting that maximized the scaled values of IC_50_ of both targets (spIC_50_ (EGFR) x spIC_50_ (PI3Kp110β) ≥ 0.1) and assumed LogBB ≥ −0.2 (Figure 3). Most molecules were predicted as weakly BBB permeant.

Of the 52 identified molecules, 14 were found present in the ChEMBL database, meaning that about 73.1% correspond to new molecules without any information about their activity in potential targets.

### 3.3. Self- and Cross-Docking Analysis Supported High Correlation between Activity Values and Unbiased Docking Score Using Optimal 3D-Models

**Figure 4:**
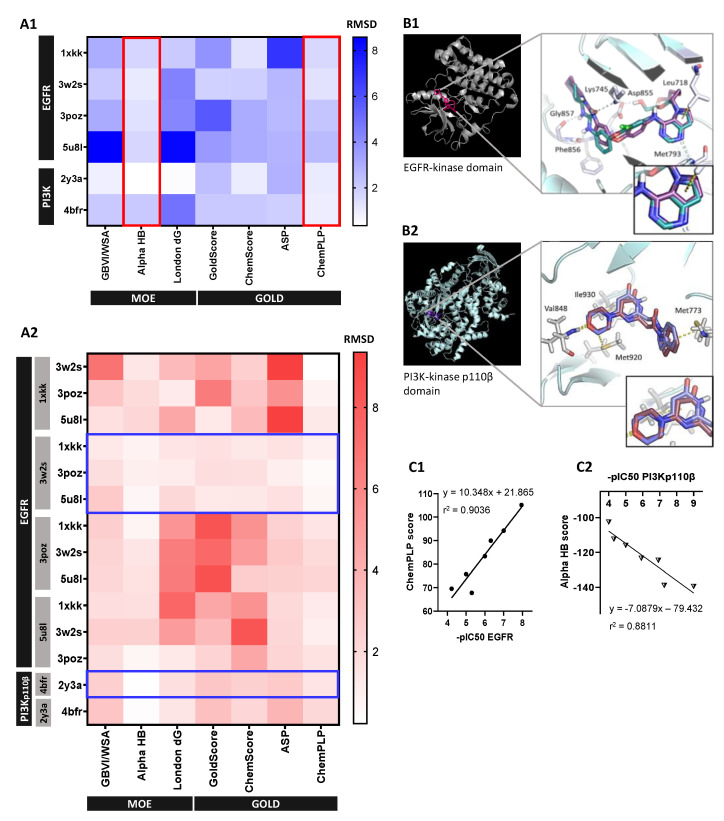
Selection and validation of unbiased molecular docking protocol: (**A**) Identification of most-fitted score function and crystallographic structure for each protein system by (**A1**) self-docking (plotted by red lined boxes) and (**A2**) cross-docking simulations (plotted by blue lined boxes), respectively. Heat maps are representative of RMSD values (in Angstrom) between the best-scoring docked pose and the co-crystallized ligand for each available crystallography, where each row represents a ligand and the columns the scoring functions for each tested software, MOE and GOLD. Grey boxes at left in (**A2**) represent the crystal structure used for each row ligand docking. (**B**) Protein–ligand interactions analysis. Ribbon representation of final (**B1**) EGFR (3w2s) and (**B2**) PI3Kp110β (4bfr) 3D structures with close-up view of the relevant binding residues using cartoon visualization of representative alignment of docked ligand (red) and crystallographic ligand (blue) from self-docking. (**C**) Validation of selected 3D models and docking protocols by correlation analysis between experimental values of standard molecules -pIC_50_ and (**C1**) GOLD CHEMPLP score for EGFR 3w2s 3D-model and (**C2**) MOE Alpha HB score for PI3Kp110β 4bfr 3D-model. Correlation coefficients r^2^ were obtained by linear regression statistical analysis.

Self-docking results showed that MOE AlphaHB and GOLD ChemPLP scoring functions reproduced the co-crystallized ligands more accurately across both EGFR and PI3Kp110β structures (RMSD < 2.5 Å) (Figure 4A1). For those protocols, the most representative 3D-models were based on PDBID:3w2s (EGFR) and PDBID:4bfr (PI3Kp110β) structures, assuming RMSD values between docked and X-ray poses of 1.251 and 0.342 Å, respectively. These findings were supported by cross-docking results since 3w2s and 4bfr were the structures able to accommodate the higher number of ligands with a pose overlapping the co-crystallized one (Figure 4A2). MOE AlphaHB and GOLD ChemPLP functions also showed the best results in cross-docking simulations both on scoring and ranking power, by reproducing the crystallographic pose (0.24–2.45 Å and 0.22–2.20 Å RMSD ranges, respectively) and displaying the highest correlation between score and IC_50_ values. Considering the protein–ligand interactions, these docking protocols successfully reproduced the consensus H-bonding residues involved in EGFR- and PI3-kinase domain-binding interface, namely, the residues Lys745, Asp855, Leu718, Met793, Phe856, and Gly857 for EGFR (Figure 4B1), and Val848, Ile930, Met773, and Met92 for PI3Kp110β (Figure 4B2).

Validated docking protocols consistently correlated standard molecules’ -pIC_50_ values, within a representative range, with GOLD ChemPLP score for EGFR 3w2s structure and MOE AlphaHB score for PI3Kp110β 4bfr structure, assuming a linear correlation coefficient r^2^ of 0.9036 (Figure 4C1) and 0.881 (Figure 4C2), respectively.

### 3.4. Unbiased in Silico Testing Supported Experimental Testing of 27 Molecules

The 52 screened molecules from ZINC15 were reduced to 27 molecules of interest, including 18 EGFR inhibitors (molecules 1–18), 6 PI3Kp110β inhibitors (molecules 19–24), and 3 dual inhibitors (molecules 25–27). The most relevant information about those molecules is summarized in Table 1, namely, regarding the QSAR model’s predictions, and their docking profiling.

Overall selected molecules have good values of predicted BBB transport and molecular activity in respective targets. EGFR candidates assumed higher values of logBB ranging from 0.203 to 0.571 when compared to PI3Kp110β (0.0097–0.162) or dual target (−0.0718 to −0.156) candidates. However, when comparing the values of -pIC_50_ (>7) with the docking binding energies, we observed lower score values for EGFR candidates than would be expected. Even though, all EGFR candidates with low binding energies (<60) assumed a high ligand efficiency (>3). Overwhelming, PI3Kp110β candidates performed much better than the other candidates in docking simulations fitting the pocket of the target with the lowest energy resources probably due to their flexible chains.

### 3.5. ADMET Properties Assessment Supported Molecules’ Drug-Likeness

For the 27 selected molecules, most descriptors in silico estimated by QikProp were within the range of values for 95% of known drugs (Table 1). Above 78% of the molecules naturally exhibit great pharmacological properties according to the Lipinski rule and are more likely to be membrane permeable and easily absorbed via passive diffusion in the human intestine. The molecular weight (MW) of considered molecules was below 600 Da, where about 74% presented optimal values under 400 Da.

All molecules were predicted to have central nervous system (CNS) activity only assuming passive BBB diffusion, except for one molecule (CNS activity: −2). Regarding octanol/water partition coefficient (QPlogPo/w) calculations, almost all candidates were at the higher range of the recommended values (>4), which translates to optimal lipidic solubility and high passive diffusion ability. All molecules showed low propension for binding human serum albumin (QplogK_hsa_) and forming non-BBB permeable complexes (QplogS). The apparent gut–blood barrier diffusional ability was also predicted (QPPCaco), supporting great absorption properties (>500 nm/s). All molecules presented a low number of reactive functional groups (#rtvFG), which can lead to false positives in screening assays and to decomposition, reactivity, or toxicity problems in vivo, supporting the effectiveness of the PAINS filter applied.

According with the ADMETsar2.0 analysis, most molecules were pointed as free of toxic/carcinogenic secondary effects and, as such, easily absorbed orally.

For each candidate are represented the following parameters: SMILES, QSAR model’s predictions of LogBB and –pIC50 for each target; probabilistic surface of molecular activity (PMSA) in respective targets; resultant score of the validated functions of docking simulation (CHEMPLP scoring for EGFR model and Alpha HB scoring for PI3Kp110β model); and respective ligand efficiency (score/number of heavy atoms).

### 3.6. Screening Analysis of Molecules’ Cytotoxicity in GB Cells Validated In Silico Predictions and Supported Low Range EC_50_ for 6 Promising Molecules

To establish the cytotoxic profile of the selected 27 molecules, cell viability was assessed (MTT test) in both U87MG and U87MG-wtEGFR cell lines (Supplementary Figure S1). About 92% of the molecules caused a significant decrease in cell viability in at least one concentration and/or cell line. Most molecules targeting EGFR (1–18) performed better in U87MG-wtEGFR, supporting their specificity for the considered target and the higher sensitivity of this cell-line for TKI action. The cytotoxic effect observed for some molecules (e.g., 6–8), reached a plateau at very low concentrations, and it was especially evident in the U87MG-wtEGFR cell-line, suggesting some type of saturation.

**Figure 5:**
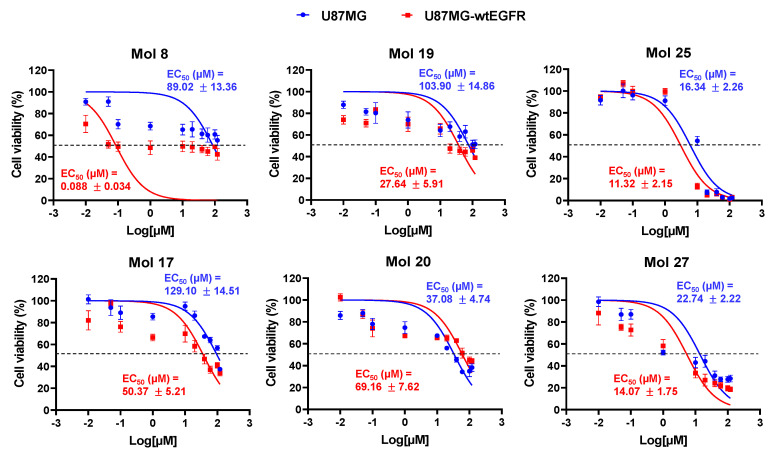
Drug-response curves delivered low-range EC_50_ values for the six most promising molecules. U87MG and U87MG-wtEGFR cells were incubated for 24 h with 10 different concentrations (0.01–120 µM) of each selected drug (8, 17, 19, 20, 25, and 27), or vehicle (control). Cell viability was assessed by MTT assay, and the values are presented as percentage relative to control. All experimental values are means ± SEM and were obtained from three independent experiments performed in triplicate.

When the threshold was set at 50% of cell viability, molecules 8, 17, 19, 20, 25, and 27 showed remarkable effects on both cell lines. For five of those molecules, the percentage of viable cells decreased in a dose-dependent manner, although the degree of cell viability impairment varied. Molecule 25 showed a remarkable cytotoxicity at 100 µM, with nearly 0% of viable cells. For the stated six molecules, additional assays were performed to establish the 24 h EC_50_ for each GB cell-line (Figure 5). Most molecules assumed lower EC_50_ values in U87MG-wtEGFR when compared with the parental cell-line U87MG. Noteworthy, dual targeting candidates assumed the lowest values of EC_50_ in both cell-lines (11–23 µM), inducing more than 80% of cell viability impairment.

### 3.7. Selected Molecules Treatment Led to Decreased EGFR and/or AKT Phosphorylation and Increased U87MG Cells Apoptosis

Activation of EGFR signaling is associated with its phosphorylation, as well as of downstream players. Thus, the expression of phosphorylated EGFR and AKT (downstream substrate of PI3K) in U87MG was analyzed by immunofluorescence after incubation with each selected molecule. Specifically, the status of T1068 residue of the EGFR-kinase domain and the Ser473 residue of AKT were analyzed.

**Figure 6:**
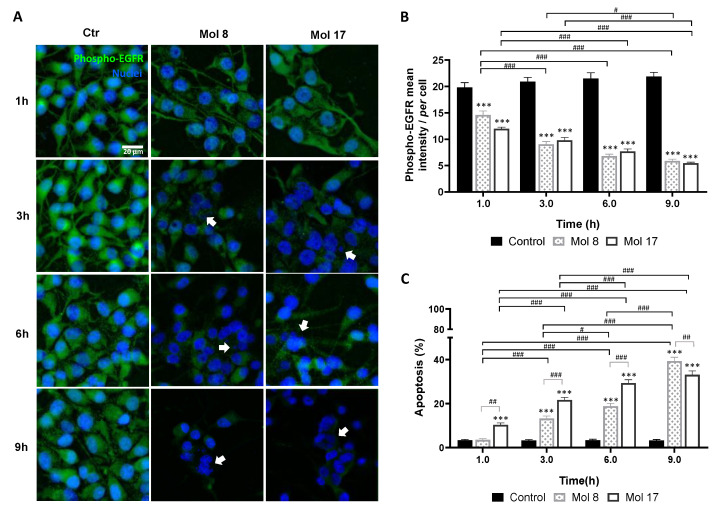
EGFR inhibitors lead to decreased phospho-EGFR expression and increased glioma cell apoptosis. U87MG cells were grown on coverslips for 48 h and then incubated with molecule (Mol) 8 or 17 at EC_50_, or vehicle (control; Ctr): (**A**) Immunostaining for phospho-EGFR and nuclei labeling with Hoechst 33342 were performed at different time-points. Images are representative of three independent experiments each with 10 random fields analyzed. (**B**) Semi-quantitative analysis of phospho-EGFR expression along time per cell. (**C**) Analysis of the percentage of apoptotic cells. Arrows in (**A**) point to cells that presented characteristic morphological changes of apoptosis such as condensation of chromatin and nuclear fragmentation. Data presented in (**B**,**C**) are means ± SEM of three independent experiments each with 10 random fields analyzed. Statistical analysis was performed by one-way ANOVA with Tukey correction. *** *p* < 0.001 vs. control of each time-point; ^#^ *p* < 0.05, ^##^ *p* < 0.01, ^###^ *p* < 0.001 between indicated groups.

For EGFR targeting molecules, 8 and 17, a relevant decrease in phospho-EGFR expression was observed after 1 h of exposure (Figure 6A), reaching about 30% of that observed in control at 9 h (*p* < 0.001, Figure 6B). Moreover, an increase in nuclei with morphological changes typical of apoptosis, such as condensation of chromatin and nuclear fragmentation, was observed along time (Figure 6A). By counting the percentage of apoptotic cells (Figure 6C), both molecules induced almost 40% of cell death at 9 h (*p* < 0.001). Even though, molecule 17 seems to have a faster anti-tumor effect by causing more apoptosis at early stages (*p* < 0.001, 1 h) with statistical differences between treatments observed until 6 h (*p* < 0.01, 1 h; *p* < 0.001, 3 h and 6 h).

**Figure 7:**
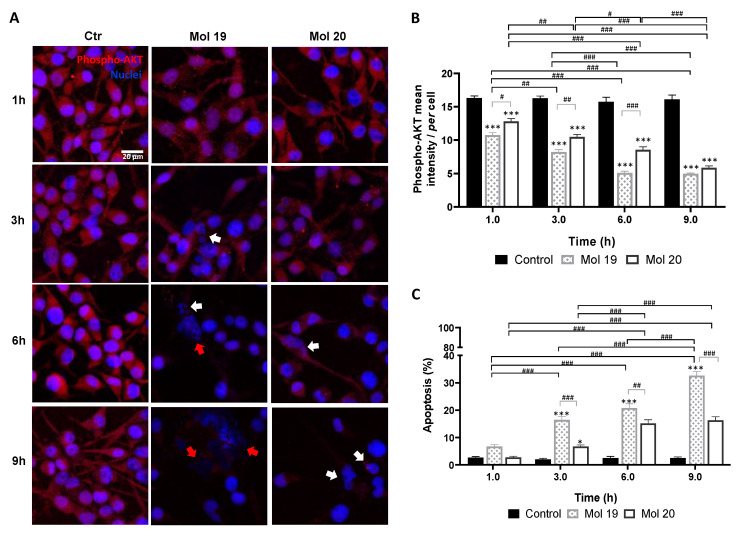
PI3Kp110β inhibitor candidates lead to decreased phospho-AKT expression and increased glioma cell apoptosis. U87MG cells were grown on coverslips for 48 h and then incubated with molecule (Mol) 19 or 20 at EC_50_, or vehicle (control; Ctr): (**A**) Immunostaining for phospho-AKT and nuclei labeling with Hoechst 33342 were performed at different time-points. Images are representative of three independent experiments each with 10 random fields analyzed. (**B**) Semi-quantitative analysis of phospho-AKT expression along time per cell. (**C**) Analysis of the percentage of apoptotic cells. White arrows in (**A**) point to cells presenting characteristic morphological changes of apoptosis such as condensation of chromatin and nuclear fragmentations, whereas red arrows point to necrotic-like areas with consistent nuclear lysis. Data presented in (**B**,**C**) are means ± SEM of three independent experiments, each with 10 random fields analyzed. Statistical analysis was performed by one-way ANOVA with Tukey correction. * *p* < 0.05, *** *p* < 0.001 vs. control of each time-point; ^#^ *p* < 0.05, ^##^ *p* < 0.01, ^###^ *p* < 0.001 between indicated groups.

Analysis of phosphorylated AKT revealed that molecules 19 and 20 lead to a decreased expression as compared with the control (Figure 7A), indicating that these molecules effectively inhibited AKT signaling pathway. Semi-quantitative analysis of the labeling intensity (Figure 7B) showed a prevailing effect of molecule 19 (*p* < 0.05, 1 h; *p* < 0.01, 3 h; *p* < 0.001, 6 h)**,** indicating a faster action on the target than molecule 20. This translated into a higher apoptotic cell death (Figure 7C), achieving about 35% at 9 h (*p* < 0.001), as compared with about 15% observed for molecule 20 (*p* < 0.001). Moreover, molecule 19 not only caused cell shrinkage and apoptotic bodies, but also caused nuclear blebbing and membrane lysis at later stages, consistent with an inflammatory process possibly delivered by necrosis (Figure 7A, 6–9 h).

**Figure 8:**
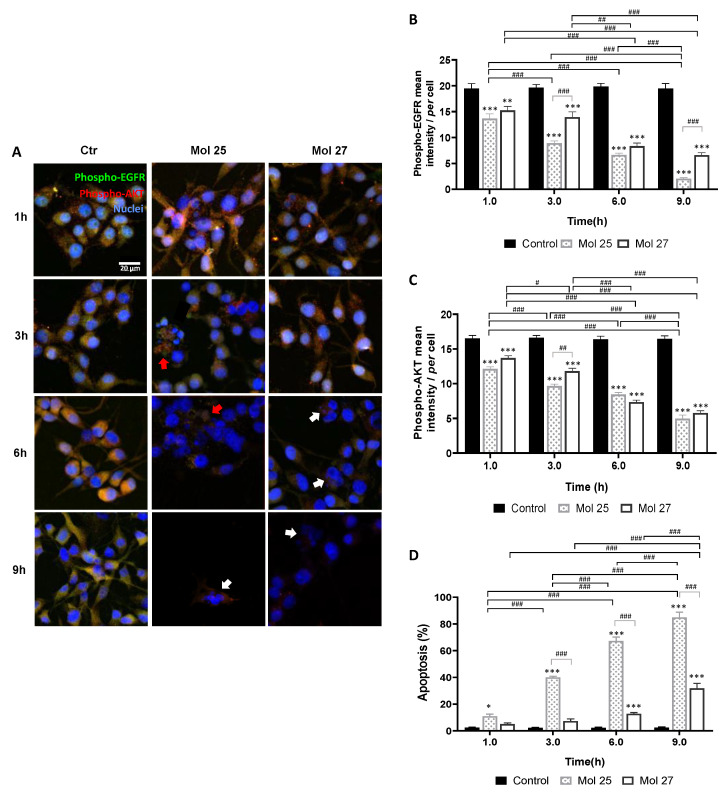
Dual targeting candidates lead to decreased phospho-EGFR and -AKT expression and increased glioma cell apoptosis. U87MG cells were grown on coverslips for 48 h and then incubated with molecule (Mol) 25 or 27 at EC_50_, or vehicle (control; Ctr): (**A**) Double immunostaining for phospho-EGFR and phospho-AKT were performed at different time points. Nuclei were counterstained with Hoechst 33342. Images are representative of three independent experiments each with 10 random fields analyzed: (**B**) Semi-quantitative analysis of phospho-EGFR expression along time per cell. (**C**) Semi-quantitative analysis of phospho-AKT expression along time per cell. (**D**) Analysis of the percentage of apoptotic cells. White arrows in (**A**) point to cells presenting characteristic morphological changes of apoptosis such as condensation of chromatin and nuclear fragmentations, whereas red arrows point to necrotic-like areas with consistent nuclear lysis. Data presented in (**B**–**D**) are means ± SEM of three independent experiments, each with 10 random fields analyzed. Statistical analysis was performed by one-way ANOVA with Tukey correction. * *p* < 0.05, ** *p* < 0.01, *** *p* < 0.001, vs. control of each time-point; ^#^ *p* < 0.05, ^##^ *p* < 0.01, ^###^ *p* < 0.001 between indicated groups.

The effect of the dual targeting molecules, 25 and 27, in both phospho-EGFR and -AKT, was also assessed by double-labeling immunofluorescence analysis (Figure 8). Immunofluorescence analysis indicated cell membrane alterations, noted by the rounded cell shape, detachment from the substrate, and fragmentation predominantly after 6 h incubation (Figure 8A). Likewise, analysis of phospho-EGFR and phospho-AKT immunostaining intensity along time (Figure 8B,C, respectively) suggests that both molecules inhibit the phosphorylated target expression mainly after 6 h exposure (>50%, *p* < 0.001). Moreover, molecule 25 appears more specific for EGFR phosphorylation inhibition than molecule 27 (*p* < 0.001, 3 h and 9 h), reducing the mean intensity of its phosphorylated form in 92.6% vs. 66.5% at 9 h (*p* < 0.001).

Regarding apoptotic cell death (Figure 8D), a 12.8% increase at 6 h (*p* < 0.001) and a further rise to 31.9% at 9 h (*p* < 0.001) were observed for molecule 27. Although, the anti-tumor effect of molecule 25 was groundbreaking when compared with molecule 27-induced cell death (*p* < 0.001, 3–9 h), reaching almost 85% of apoptosis after 9 h exposure (*p* < 0.001), which supports the previous screening results. Seemingly to molecule 19, molecule 25 induced several necrosis-like phenotypical markers. The effects of molecule 25-treatment occur in a short time window since apoptotic bodies and cell lysis were predominantly observed at 3 h and 6 h, whereas at 9 h almost no cells were found and the few that remained were clearly apoptotic (Figure 8A).

These results validate the in silico predictions by showing that molecules with predicted ability to inhibit EGFR indeed compromise its activation as indicated by the decreased expression of the phosphorylated form of the receptor, and that those identified as inhibitors of PI3K reduce the activation of its downstream signaling molecule, AKT, as shown by the decreased expression of the phosphorylated form. Moreover, they showed that the dual inhibitors block the activation of both EGFR and PI3K signaling. Collectively, these results demonstrate the reliability of the QSAR model to reveal novel drug candidates for inhibition of EGFR and PI3K activation for a multitargeting therapeutics for GB.

### 3.8. Analysis of HBMEC Junctional Tightness, Morphology, and Efflux Activity Supported three Candidates as Tailored for Brain Delivery

**Figure 9:**
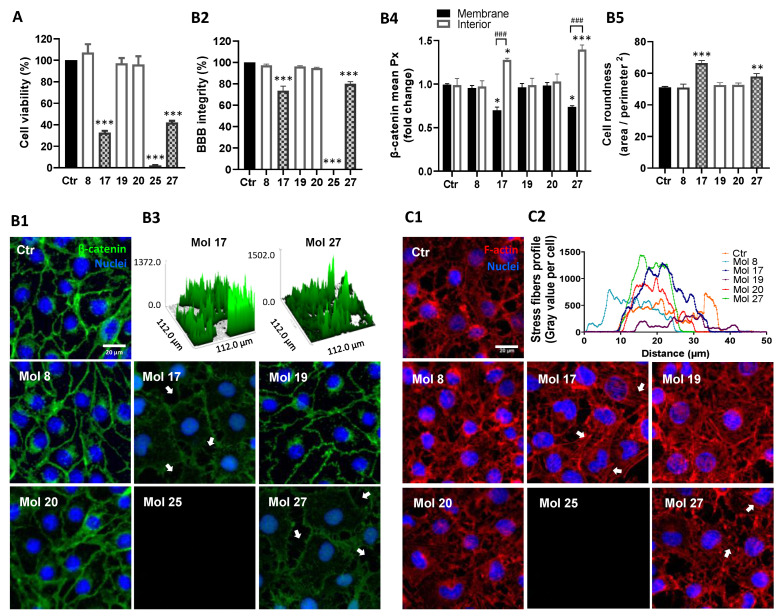
HBMEC treatment with selected molecules proved lack of cytotoxicity for 3 candidates. HBMEC were exposed to each of the selected molecules (8, 17, 19, 20, 25, and 27), or no addition (control; Ctr), at a concentration of 100 µM, for 24 h, for viability assays (**A**), or at U87MG EC_50_, during 9 h, for BBB integrity (**B**) or cytoskeleton (**C**) analyses. (**A**) Cell viability was assessed by MTT assay and the values are presented as a percentage relative to control. Molecules with significant cytotoxic effects are presented as pattern-filled bars, and innocuous molecules are shown as white bars. (**B**) BBB integrity was assessed by immunostaining of β-catenin, with nuclear counterstaining by Hoechst 33342. (**B1**) Arrows point to gaps in the HBMEC monolayer caused by incubation with toxic molecules. (**B2**) Semi-quantitative analysis of endothelial gaps, where values are presented as percentage of control. (**B3**) 3D representative plots of β-catenin expression obtained by counting the number of image pixels per area in disrupted monolayers. (**B4**) Semi-quantitative analysis of β-catenin internalization by fold-change of mean image pixels (Px) in the membrane and interior of treated cells vs. control. (**B5**) Morphological analysis of HBMEC roundness by cells delineation. (**C**) Cytoskeleton analysis was performed based on F-actin labeling. (**C1**) Arrows highlight the presence of stress fibers. (**C2**) Cytoskeleton stress fibers profile per cell representation by semi-quantitative analysis of gray values expressed along ferret diameter of each cell. Images are representative of three independent experiments each with 10 random fields analyzed. All values in (**A**) are means ± SEM of three independent experiments performed in triplicates. Semi-quantitative analyses shown in (**B**) are means ± SEM of three independent experiments. Statistical analysis was performed by one-way ANOVA with Tukey correction. * *p* < 0.05, ** *p* < 0.01, *** *p* < 0.001 vs. control; ^###^ *p* < 0.001 between indicated groups.

MTT cell viability assay (Figure 9A) revealed a lack of cytotoxicity of molecules 8, 19, and 20 in HBMEC at a concentration higher than the EC_50_ (100 µM). Contrarywise, molecules 17, 25, and 27 showed cytotoxicity, impairing cell viability in about 65%, 100%, and 58%, respectively (*p* < 0.001).

The junctional protein β-catenin expression was also analyzed to understand the effects of the molecules at EC_50_ on endothelium integrity (Figure 9B). In control cells, β-catenin exhibited a continuous line at the cell–cell contacts; however, after molecule 17 or 27 treatment, this junctional protein assumed an irregular and discontinuous appearance (Figure 9B1). Molecules 17 and 27 disrupted barrier integrity (78% and 82% of control, *p* < 0.001, respectively, Figure 9B2), increasing the number and size of monolayer gaps (Figure 9B3). Moreover, the distribution of β-catenin evaluated via densitometric analysis (Figure 9B4), showed that after molecules 17 and 27 treatments HBMEC exhibited more cytoplasmatic (*p* < 0.05 and *p* < 0.001, respectively) and less membrane β-catenin (*p* < 0.05) compared to controls, supporting a higher internalization of β-catenin and consequent compromise of barrier properties. Simultaneously, the results supported a high cytotoxic effect of molecule 25, inducing 100% of HBMEC detachment (Figure 9B1,B2), and the absence of changes in β-catenin expression and location by the treatment with molecules 8, 19, and 20.

After a morphological study (Figure 9B5), no significant changes in HBMEC shape were detected by molecules 8, 19, and 20 treatments. Contrarywise, molecules 17 and 27 increased cell roundness (*p* < 0.001 and *p* < 0.01, respectively). The morphological changes in HBMEC after the molecules treatment were also considered by F-actin cytoskeleton protein expression analysis (Figure 9C1). Consistently with previous results, molecules 17 and 27 highly increased the number of stress fibers/cell, especially at the ventral cell surface, but molecule 20 also caused a slight intensification relative to the control baseline (Figure 9C2). Molecule 25 again proved its high cytotoxicity by inducing all-cells detachment (Figure 9C1).

Knowing the influence of efflux transporters in brain drug delivery, the expression of P-gp in HBMEC was also analyzed (Supplementary Figure S2). According to prior in silico ADMET predictions, molecules 17 and 27 were pointed out as being more likely substrates of P-gp, which was validated by a significant increase in P-gp mean intensity after molecules treatment (*p* < 0.05 and *p* < 0.001, respectively). No significant changes in P-gp expression were detected after molecule 8, 19, and 20 treatments.

Overall, molecules 17, 25, and 27 were cytotoxic for HBMEC, while molecules 8, 19, and 20 were safe for direct drug delivery, thus proceeding to further assays of BBB transport.

### 3.9. Selected Molecules Proved to Efficiently Cross the BBB Reinforcing In Silico Predictions

**Figure 10:**
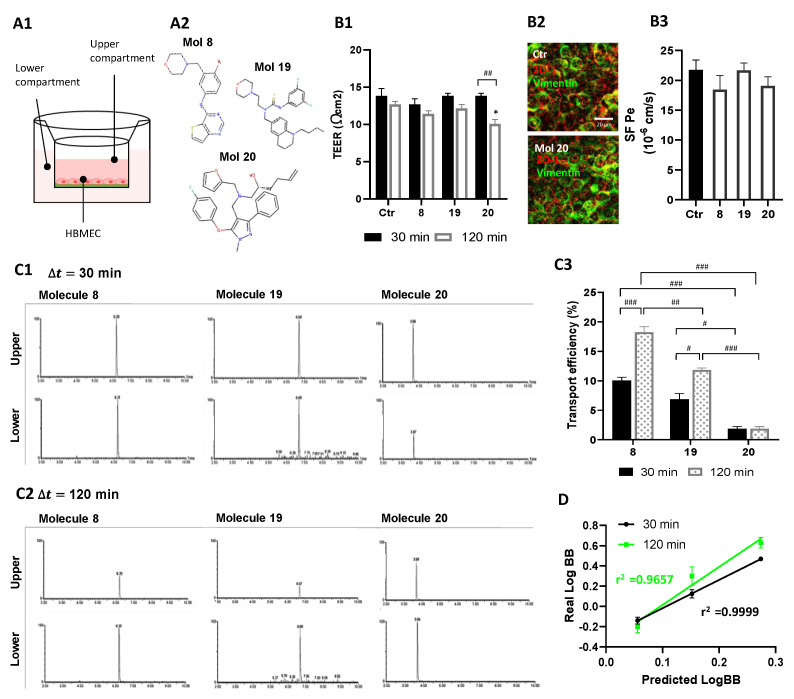
Selected molecules proved to efficiently cross the BBB. (**A**) HBMEC were grown to confluence in Transwell inserts and then incubated with molecules 8, 19, or 20 at U87MG EC_50_, or vehicle (control; Ctr), for 30 or 120 min. After that, (**B**) inserts were removed and tested for barrier integrity, and (**C**) cell medium from upper and lower chambers were collected and analyzed by UPLC-MS/MS to assess molecule permeation across HBMEC. (**A1**) Schematic view of the two-chamber BBB model and (**A2**) molecular structure of the candidates tested for BBB permeation. (**B1**) BBB integrity was assessed by TEER measurement. (**B2**) Representative immunostaining for HBMEC expression of ZO-1 and vimentin, junctional, and cytoskeleton proteins, respectively. (**B3**) Quantitative analysis of the permeability to SF (SF Pe) across HBMEC in the Transwell system. (**C**) Total Ion Chromatograms (TIC) of MRM analyses obtained by UPLC-MS/MS. Quantification was based on the integration (peak areas) of the represented well-resolved peaks with reproducible retention times, both in upper and lower compartments at (**C1**) 30 min and (**C2**) 120 min of incubation with molecule 8, 19, or 20. (**C3**) Transport efficiency quantification is expressed as a percentage of initial applied concentration. (**D**) Linear correlation between predicted and experimentally validated values of LogBB, expressing the degree of BBB permeation. Statistical analysis was performed by one-way ANOVA with Tukey correction. Data presented in (**B**) are means ± SEM of six independent experiments and in (**C**) of three independent experiments. * *p* < 0.05 vs. control; ^#^ *p* < 0.05, ^##^ *p* < 0.01, ^###^ *p* < 0.001 between indicated groups. Correlation coefficients r^2^ (**D**) were obtained by linear regression statistical analysis.

The Transwell-based in vitro BBB model was used (Figure 10A1) to assess the integrity of the HBMEC monolayer during assays with molecules 8, 19, and 20 (Figure 10A2). None of the molecules markedly changed the TEER at 30 min of exposure, but unexpectedly molecule 20 caused a TEER decrease at 120 min (*p* < 0.05; Figure 10B1). This TEER decrease did not cause barrier leakiness as demonstrated by immunostaining for ZO-1 and vimentin, junctional, and cytoskeleton proteins, respectively (Figure 10B2). Similarly, analysis of HBMEC permeability to sodium fluorescein (Figure 10B3) indicated no statistical differences between treated cells and control, presenting an index of paracellular diffusion (Pe) in line with previous findings [37].

Taking advantage of the validated in vitro model of the human BBB, we confirmed by UPLC tandem mass spectrometry that molecules 8, 19, and 20 could be transported across the BBB endothelium. Results show the different retention times and the well-resolved peaks, where respective integration of peak areas (PA) enabled to estimate the concentration of each molecule in both chambers either after 30 or 120 min incubation (Figure 10C1,C2). These data suggest similar levels of molecules 8 and 19 in upper and lower chambers at 30 min, supporting a higher BBB permeation when compared to molecule 20. Moreover, we observed that at 120 min, molecules 8 and 19 were mostly found in lower compartments, whereas such a difference was less evident for molecule 20. Interestingly, a trend of an overall decrease in time of total PA in both compartments was noted, suggesting that HBMEC can metabolize a fraction of the molecules (data not shown).

Analysis of endothelial transport efficiency showed that molecules 8 and 19 are transported across the HBMEC monolayer in a time-dependent manner (*p* < 0.001, molecule 8; *p* < 0.05, molecule 19, 30 vs. 120 min), while molecule 20 transport did not increase significantly from 30 to 120 min of incubation (Figure 10C3). All molecules proved a higher-off range transport relatively to well-known BBB permeable compounds.

LogBB values were also calculated as a measure of brain penetration extent. Mean values of LogBB experimentally obtained at 30 and 120 min were impressively correlated with in silico predictions (Table 1), translated by high linear correlation coefficients (Figure 10D).

### 3.10. Selected Molecules Crossed the BBB Endothelium and Induced Cytotoxicity in GB Cells in a Representative Co-Culture Model

**Figure 11:**
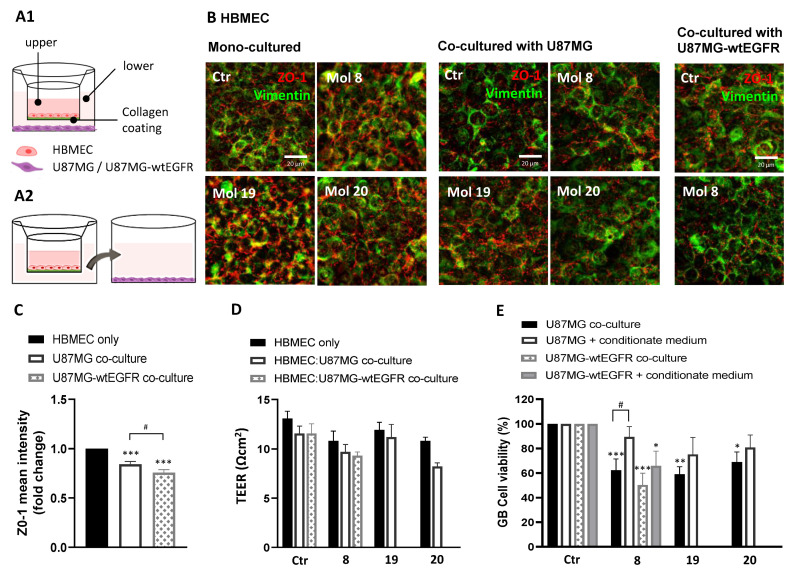
A reliable co-culture system corroborated candidate molecules as BBB permeable and strong anti-GB agents: (**A**) HBMEC were grown to confluence in Transwell inserts and U87MG or U87MG-wtEGFR cells were seeded in the lower chamber. After 3 days of co-culture, supernatant in the upper chamber was replaced with fresh medium (control) or with medium supplemented with molecules 8, 19, or 20 at EC_50_. After 2 h, the inserts were removed and tested for barrier integrity (**B**–**D**), whereas the gliomasphere-like cells were maintained in culture for more than 24 h to assess cell viability by MTT assay (**E**). In parallel, the U87MG or U87MG-wtEGFR cells were incubated for 24 h with the basolateral conditioned medium of HBMEC after incubation with the molecules. (**A1**) Schematic representation of the BBB-GB co-culture model and (**A2**) of the conditioned medium assay. (**B**) Immunostaining for ZO-1 and vimentin in HBMEC mono-culture or co-cultured with U87MG or U87MG-wtEGFR. Images are representative of three independent experiments each with 10 random fields analyzed. (**C**) Semi-quantitative analysis of ZO-1 expression in HBMEC cells mono-cultured vs. co-cultured with U87MG or U87MG-wtEGFR by mean fluorescence intensity fold-change. (**D**) TEER of HBMEC after 2 h of incubation with the molecules both in mono- or co-culture system. (**E**) U87MG or U87MG-wtEGFR cell viability assessment by MTT assay after molecules exposure either in co-culture system or by conditioned medium. Data presented in (**C**,**E**) are means ± SEM of three independent experiments each with 10 random fields analyzed. Values in (**D**) are means ± SEM of three independent experiments. Statistical analysis was performed by one-way ANOVA with Tukey correction. * *p* < 0.05, ** *p* < 0.01, *** *p* < 0.001; ^#^ *p* < 0.05 between indicated groups.

Molecules’ transport and cytotoxicity were assessed in an archetypical BBB-GB co-culture model, implemented in our laboratory (Figure 11A1), and through a basolateral conditioned experiment (Figure 11A2). Since molecule 8 is specific for EGFR inhibition, its effect was analyzed both in HBMEC:U87MG and HBMEC:U87MG-wtEGFR co-culture systems, while, molecules 19 and 20 only were evaluated in HBMEC:U87MG co-culture. The drugs were added to the “blood side” and their effect in GB cells in the “brain side” was assessed following 2 h of incubation, a timepoint at which drugs were able to cross the BBB endothelium (Figure 10).

Compared to HBMEC mono-cultured, the presence of U87MG or U87MG-wtEGFR induced a slight decrease in barrier tightness, indicated by the decreased staining for ZO-1 (Figure 11B). Semi-quantitative analysis of ZO-1 mean intensity (Figure 11C) revealed a decrease in the co-culture system free of drug treatment (*p* < 0.001), especially in the presence of U87MG-wtEGFR (*p* < 0.05). As expected, TEER readings were not decreased by molecules 8, 19, and 20, supporting their non-cytotoxic profile for HBMEC (Figure 11D), and corroborated by the unaltered ZO-1 immunostaining (Figure 11B). In line with ZO-1 expression, a slight TEER decrease in HBMEC co-incubated with GB cells vs. mono-cultured was observed, although not significant.

The three molecules impaired U87MG B cell viability in the co-culture system (*p* < 0.001, molecule 8; *p* < 0.01, molecule 19; *p* < 0.05, molecule 20; Figure 11E), and Mol8 also impaired U87MG-wtEGFR in co-cultures (*p* < 0.001), confirming their BBB permeation. However, none of the molecules induced a statistically significant difference in cell viability of U87MG incubated with basolateral conditioned medium of HBMEC in the BBB model, with a significant difference only observed for U87MG-wtEGFR incubated with molecule 8 (*p* < 0.05). The marked decrease in GB cell viability in the co-culture system suggests that GB cells release soluble factors that increase BBB’s permeability, which may turn into an advantage for drug delivery for GB treatment. Overall, these studies indicate that the studied drug candidates may permeate across the BBB endothelium and reach therapeutic concentrations at the target GB cells in the “brain side” of the experimental system.

## 4. Discussion

An automated and extendable platform based on our previous work [20] was used as a QSAR modeling pipeline to obtain optimized predictive models for three different chemical problems, namely overcoming the BBB and targeting EGFR and PI3Kp110β signaling.

The obtained results of the developed BBB permeation QSAR model largely exceed the quality of the most recent published empirical models, most of which lack biological validation [39,40]. Still, the developed BBB’s permeation model is especially better in predicting compounds that can cross the BBB than the non-permeable ones, which makes it more tailored to be incorporated in drug discovery programs for brain delivery than non-CNS contexts. Concerning EGFR and PI3Kp110β inhibition models, they represent pioneering efforts in the data mining field since no targeted drug for brain tumors elected by regression QSAR models met the conditions to achieve clinical trials. Importantly, no QSAR model predicting the specific inhibition of PI3K subunit p110β has ever been built, because most efforts have focused on subunit p110α, which also supports the relevance of our models for further drug discovery programs. It is important to mention that although the identified molecules are predicted to specifically inhibit the β isoform, inhibition of the α isoform may not be disregarded. Anyway, considering that both isoforms lead to AKT activation, any contribution of the α isoform would be reflected in the phospho-AKT expression.

The produced models were used to screen the ZINC15 database. The latest version of ZINC15 accounts for more than 200 million “drug-like” molecules readily available for commercial purchase, some of which are associated with specific targets or diseases, opening new-fangled possibilities for their repurposing for novel clinical applications. From ZINC15 screening, five Food and Drug Administration (FDA)-approved compounds were found (canertinib, naquotinib, tesevatinib, poziotinib, and PKI-166), with potential to be repurposed in the GB clinical context. Moreover, the other candidates represent new options never explored before.

Allying the straightforward results from ZINC15 screening with docking studies represents a powerful strategy that has helped unravel the structural and energetic bases of the interaction between targets and candidate molecules. Overall, both EGFR and PI3Kp110β 3D-models allowed a great reproducibility of ligands binding mode and exhibit great performance in the establishment of a relationship between score and inhibitory capacity, clearly distinguishing between active and non-active compounds. By visual inspection of the molecules in the respective pocket and analysis of established bounding, we observed that most interactions were assured. These results, together with the predicted ADMET profile and drug-like phenotype of selected small molecules, strongly support the studied molecules as robust and qualified candidates for experimental testing.

The cytotoxic potential of the selected 27 molecules (18 EGFR inhibitors, 6 PI3Kp110β inhibitors, and 3 dual inhibitors) in two well-recognized GB cell lines, namely U87MG (parental) and U87MG overexpressing EGFR (U87MG-wtEGFR), was explored by cell viability analysis. Most tested molecules promoted tumor cell death, from which two EGFR inhibitors (molecules 8 and 17), two PI3Kp110β inhibitors (molecules 19 and 20), and two dual inhibitors (molecules 25 and 27) caused more than 50% of the cell death. Their respective dose–response curves fetched EC_50_ values in the 16–130 µM and 0.09–70 µM range for U87MG and U87MG-wtEGFR, respectively, which largely outperform drugs currently under investigation.

The effectiveness of the EGFR TKI gefitinib was tested in recurrent GB, but the downstream effectors remained constitutively active, leading to unsatisfactory effectiveness [41]. More recently, efforts were focused on the use of dacomitinib, an oral, irreversible, second-generation, pan-HER TKI [8], but poor global results in recurrent GB were obtained in a phase II clinical trial [42]. Moreover, most of those molecules showed poor tumor-suppressive effects in pre-clinical studies under concentrations of 100 µM [43,44], which render our EGFR candidates powerful interviewees in the current market.

Regarding PI3K inhibitors, the new generation of pan-PI3K inhibitors, such as BKM120 and PX-866, exhibit improved drug properties such as high stability and low side effects [45]. However, their use has been halted in clinical trials due to toxicity, poor pharmacodynamics, and selectivity [46]. Despite recent advances in the study of selective PI3K inhibitors, such as alpelisib and idelalisib, no clinical study is currently ongoing focusing on the inhibition of the p110β subunit. The key factor that may emerge is that although several dual PI3K/mTOR inhibitors are being investigated (XL765, PKI-587), no dual EGFR/PI3K has been known until now. Consequently, our results point to a new field of therapeutic opportunities for GB treatment. Besides all efforts to develop new medicines, the alkylating agent temozolomide (TMZ) remains the standard of care for GB patients [4]. Surprisingly, several cell-based studies have proved that TMZ dosage is far beyond the therapeutic recommended concentrations, where more than 50% cytotoxicity was only seen in up to 4000 µM doses [47,48]. Moreover, a recent systematic review of the sensitivity to TMZ of several glioma cell lines revealed the IC_50_ values in U87 cells within the 124–230 µM range, depending on the incubation time, and that the median IC_50_ at 72 h for patient-derived cell lines was 800 μM [49]. This strongly wires the potential of our drug candidates, which revealed much lower EC_50_ values. Moreover, they raise the hypothesis that a combined therapy with TMZ and one of the best drug candidates may represent a possible multitargeting therapeutic strategy for GB.

The immunofluorescence analysis supported the direct impact of the identified drug candidates in the proliferation and viability of tumor cells, translated by a reduction in the cell number and an increase in apoptotic markers along time. The key point of these results may rely on the fact that from all six selected candidates, molecule 25 (canertinib) is approved for esophageal squamous cell carcinoma, which may emerge as an actual opportunity for repurposing.

As previously mentioned, many TKIs displayed excellent in vitro efficacy but failed in pre-clinical and clinical trials because of their great toxicity and high concentrations, which results in heavy side effects and toxicities [50]. This attests the need of considering off-target effects in the drug discovery and development process. This issue was considered in the present work by including ADMET predictions in the computational studies. It was further addressed through analysis of the safety of the most promising drug candidates to resident cells. In particular, we analysed the drugs’ effects in HBMEC, considering that the focus of this work is on brain pathology and BBB permeation. Unfortunately, molecules 17, 25, and 27 showed significant cytotoxic effects against HBMEC, but they can be pursued in further studies involving GB-targeted nanomedicines, to achieve a therapeutic concentration of the drug at the intended target site, with a lower exposure in non-target organs. Contrastingly, molecules 8, 19, and 20 under the <100 µM range did not elicit toxicity, in line with the ADMET predictions. Indeed, they did not induce changes in the integrity and physiological properties of the BBB, as indicated by unaltered cell viability and morphology of endothelial cells. The results were validated by the integrity of the endothelial paracellular permeability, as shown by the absence of changes in β-catenin expression, TEER, and Pe to sodium fluorescein.

It is known that most brain targeting drugs are likely activators of BBB efflux transporters, namely, the highly expressed P-gp, which can lead to a multidrug resistance phenotype and dump any expected therapeutic effect. This is a major challenge in GB treatment since most targeted drugs developed so far are actively effluxed out of the brain, namely, some alkylating agents (e.g., TMZ), and TKI (e.g., erlotinib, dasatinib, and imatinib) [51]. In this challenging context, our results arose in accordance with the in silico predictions, where molecules 17 and 27 appeared as potential P-gp substrates by increasing its expression, while molecules 8, 19, and 20 were poorly associated with P-gp alterations, which points to them as promising choices for brain accessibility.

To evaluate BBB’s permeation of the selected molecules based on their safety to the BBB (8, 19, and 20), a well-validated Transwell-based in vitro BBB model was used [37]. The transport of these molecules across the BBB was unequivocally demonstrated within the tested concentration range, through a selective and sensitive UPLC-MS/MS method. When looking to transport efficiency results, we observed an impressive higher rate of brain penetration compared to other known BBB permeable compounds, especially for molecules 8 and 19. Curiously, molecule 20 transport revealed itself as non-time-dependent, which may reflect the involvement of other transport mechanisms subjected to transporter saturation. The chemical ability of some metabolites to establish hydrogen bonds with functional groups of biomolecules could suggest a propensity for an active transport mechanism in BBB. This was especially indicated by molecule 20 in silico descriptors, and experimental transport assay results. The chief factor may rely on the remarkable correlation between QSAR predicted values and experimental validated values of LogBB, which supports the accuracy and feasibility of our model in predicting BBB’s permeability.

In line with preceding findings, co-culture experiments proved that after exposing the luminal (“blood”) side of HBMEC to molecules 8, 19, and 20, increased delivery and cytotoxicity in GB cells growing on the “brain side” were observed compared to GB cells only incubated with the basolateral conditioned medium. These results suggest that GB cells are releasing soluble factors that may decrease BBB’s tightness and increase its permeability. Several studies have reported that vessels surrounding GB are leaky, in particular those with endothelial hyperplasia, commonly showing abnormal structural features, such as loss and/or abnormal morphology of TJs [52]. As such, it is not surprising that GB cells may have tried to subvert HBMEC cells to switch to a defective phenotype as supported by a decreased expression of ZO-1 and lower values of TEER obtained in HBMEC co-cultured with GB cells.

The overall results point to the discovered molecules as a step forward in GB treatment regarding delivery across the BBB, and strong anti-tumor activity against GB cells and non-identified collateral toxicities. Therefore, the in vivo efficacy of those molecules should be investigated in pre-clinical settings.

## 5. Conclusions

The treatment of GB requires a multidisciplinary approach that may account for the multifactorial nature of this still incurable disease. For this purpose, we developed a novel in silico approach primarily based on QSAR model contributions and docking profiling to select molecules from a large compound library against two GB targets with the potential to achieve optimal CNS exposure. The proposed translational research will expectedly emerge as an innovative multidisciplinary strategy with potential clinical application in several medical fields. In addition, by experimentally validating the developed in silico methodology as a valuable approach to speed up the drug discovery process, it can be further used in the study of any brain pathology by adapting the input parameters of the used framework, saving time and resources towards the development of novel therapeutics. Our experimental results not only threadbare a new reliable therapeutic target for GB never previously addressed, the PI3Kp110β, delivering for the first time potential dual EGFR/PI3K inhibitors. To sum up, with the work presented in this article highly promising molecules were unraveled, paving the way for repurposing headways that can upgrade the pharmaceutic market to transform GB treatment and ultimately the patients’ quality of life.

## Figures and Tables

**Table 1:** QSAR model’s predictions and docking analysis supported the selection of 27 molecules for experimental validation that predictably cross the BBB and inhibit EGFR and/or PI3Kp110β activity.

Target	MOL ID	ZINC ID	SMILES	Predicted LogBB	PMSA	Predicted –pIC50		Docking and Scoring	Ligand Efficiency
**EGFR**	1	ZINC132618	Clc1cc(Cl)cc(Nc2ncnc3ccccc23)c1	0.221	0.601	7.121		54.66	3.356
	2	ZINC955717	Fc1ccc(Nc2ncnc3ccc(Br)cc23)cc1Cl	0.221	0.8628	7.253		64.7	3.498
	3	ZINC99087	Fc1ccc(Nc2ncnc3ccc(Br)cc23)cc1	0.229	0.7229	7.397		65.51	2.786
	4	ZINC65031	Oc1ccc(Nc2ncnc3ccccc23)cc1	0.216	0.694	7.1		56.24	3.217
	5	ZINC949364	Brc1ccc2ncnc(Nc3cccc4ccccc34)c2c1	0.225	0.7371	7.243		66.25	3.551
	6	ZINC4710712	C(Nc1ccccc1-c2nnn[nH]2)c3cccnc3	0.27	0.8765	7.381		62.22	2.967
	7	ZINC2664933	COc1ccc(CNc2ncnc3sc(cc23)-c4ccccc4)cc1	0.343	0.4148	7.299		77.73	3.206
	8	ZINC71920558	Oc1ccc(Nc2ncnc3ccsc23)cc1CN4CCOCC4	0.274	0.5595	7.131		70.63	3.871
	9	ZINC13863969	C[C@@H](Nc1ncnc2ccccc12)c3ccc4ccccc4c3	0.466	0.4165	7.196		79.07	3.438
	10	ZINC9074069	CCCCc1ccc(Nc2nc(Nc3ccc(F)cc3)c4nccnc4n2)cc1	0.329	0.4019	7.46		80.78	2.786
	11	ZINC13010674	Fc1ccc(Nc2nc(NCCN3CCOCC3)nc4nccnc24)cc1	0.203	0.2197	7.184		75.64	3.465
	12	ZINC22735958	OCCN1CCN(CC1)c2nc(Nc3ccccc3)c4nccnc4n2	0.282	0.2406	7.236		74.87	3.652
	13	ZINC117048	COc1cccc(Nc2ncnc3cc(sc23)-c4ccccc4)c1	0.571	0.5189	7.401		77.21	3.217
	14	ZINC955103	Clc1cccc(Nc2ncnc3ccc(I)cc23)c1	0.282	0.6762	7.207		63.2	2.893
	15	ZINC122234	Cc1cccc(Nc2ncnc3ccc(Br)cc23)c1	0.296	0.8253	7.379		64.5	3.365
	16	ZINC140100	Brc1ccc2ncnc(Nc3ccccc3)c2c1	0.285	0.8092	7.14		62.18	3.762
	17	ZINC144105	Brc1ccc(Nc2ncnc3ccccc23)cc1	0.216	0.8074	7.1		58.15	3.994
	18	ZINC48331888	CCc1cccc(Nc2ncnc3cc(Cl)ccc23)c1	0.22	0.5624	7.16		66.02	2.634
**PI3K** **p110** **β**	19	ZINC20729292	CCCCN1CCCc2cc(CN(CCN3CCOCC3)C(=S)Nc3cc(F)cc(F)c3)ccc21	0.152	0.8860	6.797		−146.243	4.228
	20	ZINC36307506	C=CCC[C@@H](O)CN(Cc1ccco1)Cc1c(-c2ccccc2)nn(C)c1Oc1ccc(F)cc1	0.056	0.8276	6.763		−145.661	4.12
	21	ZINC9873787	CN(Cc1ccc(N2CCOCC2)cc1)C(=O)c1cc(-c2ccccc2)c(N2CCOCC2)s1	0.049	0.3576	6.774		−145.661	3.593
	22	ZINC977288	C=CCn1c(C)c(C=C2C(=O)OC3(CCCCC3)OC2=O)c2ccccc21	0.162	0.7526	6.883		−115.83	3.557
	23	ZINC68202727	CCc1c(O)cc(O)c(C(=O)c2ccc(OCCN3CCOCC3)c(OC)c2)c1CC(=O)N(CCOC)CCOC	0.0097	0.2639	6.86313		−153.535	3.534
	24	ZINC218287337	COC(=O)C[C@@H](c1oc(CN2CCCCC2)cc(=O)c1O)c1c(F)cccc1Cl	0.043	0.2736	6.913		−167.605	4.061
**EGFR** **+** **PI3K** **p110** **β**	25	ZINC27439698	Fc1ccc(Nc2ncnc3cc(OCCCN4CCOCC4)c(NC(=O)C=C)cc23)cc1Cl	−0.056	0.7359	EGFR	8.348	85.04	2.501
						PI3K	6.22	−141.013	4.147
	26	ZINC20615563	Oc1c(Cl)cc(Cl)c2ccc(NN=C3c4cc(OCC=C)ccc4-c5ccc(OCC=C)cc35)nc12	−0.122	NA	EGFR	6.42	78.9	2.744
						PI3K	6.696	−120.816	3.958
	27	ZINC1488208	Brc1cccc(Nc2ncnc3ccc(NC(=O)C=C)cc23)c1	−0.0718	0.8166	EGFR	8.796	68.3	2.972
						PI3K	5.636	−98.6951	3.866

Each ZINC_ID was associated with a MOL_ID to facilitate command tracking.

## Data Availability

All data generated or analyzed during this study are included in this published article and its Supplementary Information Files.

## Supplementary Materials

The following supporting information can be downloaded at: https://www.mdpi.com/article/10.3390/cancers14143506/s1, Figure S1: High-throughput analysis of in silico selected molecules induced cell viability impairment in U87MG and U87MG-wtEGFR cell lines allowed the selection of six highly cytotoxic candidates, Figure S2. HBMEC efflux activity points to molecules 17 and 27 as substrates of P-gp while molecules 8, 19, and 20 were not associated with an active efflux out of the brain, Table S1: Description of training datasets for each selected chemical problem, Table S2: Description of crystallographic structures selected from PDB for each protein target.
