# Supplementary material for: Multi-Targeting Approach in Glioblastoma Using Computer-Assisted Drug Discovery Tools to Overcome the Blood–Brain Barrier and Target EGFR/PI3Kp110β Signaling
Source: Cancers (Basel). 2022 Jul 19;14(14):3506. doi: 10.3390/cancers14143506 (PMC9317902; doi:10.3390/cancers14143506)
Supplement: Supplementary file 1 [file cancers-14-03506-s001.zip › cancers-1823124-supplementary.pdf]

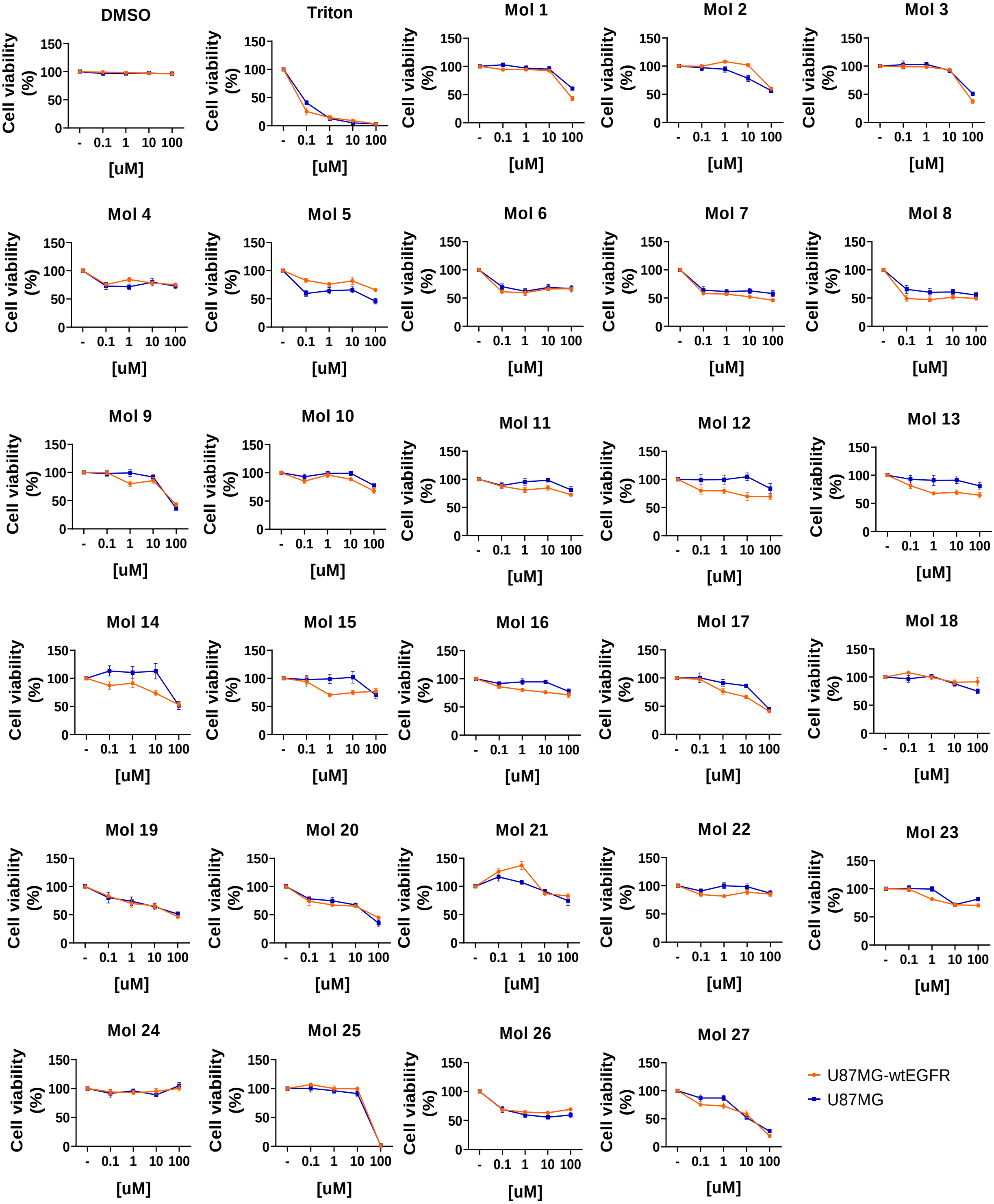

Supplementary Figure S1. Cell viability analysis of U87MG and U87MG-wtEGFR cell lines treated with *in silico* discovered molecules (Mol) allowed the selection of six highly cytotoxic candidates. Both U87MG (blue) and U87MG-wtEGFR (orange) cell lines were incubated for 24 h with 0.1, 1, 10 or 100 µM of EGFR inhibitors (Mol 1-18), PI3Kp110β inhibitors (Mol 19-24), dual target inhibitors (Mol 25-27), or vehicle (control). Cell viability was assessed by MTT assay and the values are presented as percentage relatively to the control. Mol 8, 17, 19, 20, 25 and 27 were selected for further studies since they caused ≥ 50% of cell death in both cell lines. All values are means ± SEM of three independent experiments performed in triplicate.

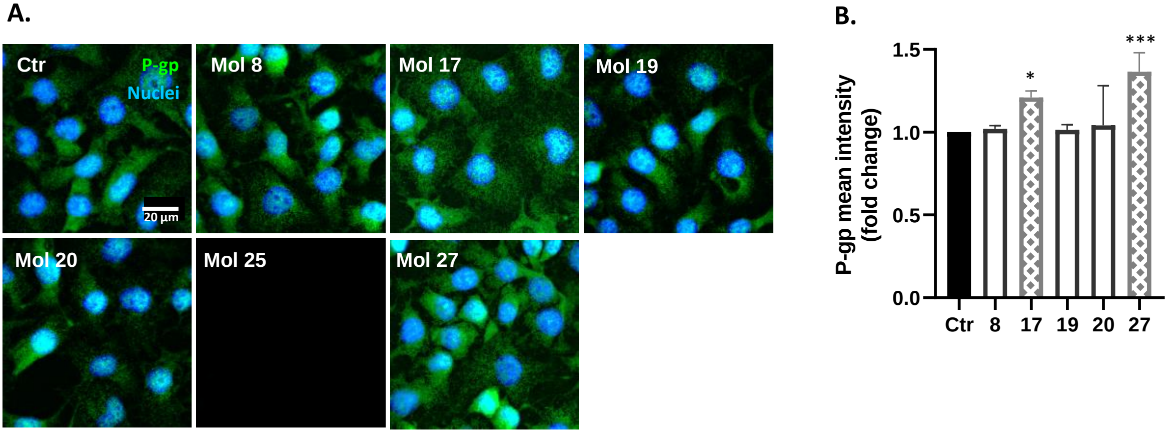

Supplementary Figure S2. HBMEC efflux activity point to molecules 17 and 27 as substrate of P-gp while molecules 8, 19 and 20 were not associated with an active efflux out of the brain. HBMEC were incubated with selected molecules (8, 17, 19, 20, 25 and 27) at U87MG EC_{50}, or vehicle (control). **(A)** Immunostaining of HBMEC for P-gp and nuclei labeling with Hoescht 33342 after 9h of incubation. Images are representative of three independent experiments each with 10 random fields analyzed. **(B)** Semi-quantitative analysis of P-gp expression in treated cells *vs.* control by fluorescence mean intensity fold-change. Increased P-gp expression is represented by pattern-filled bars. Data are presented as means ± SEM of three independent experiments. The statistical analysis for all experiments was performed by one-way ANOVA with Tuckey correction. *P<0.05, ***P<0.001 vs. control.

Supplementary Table S1. Description of training datasets for each selected chemical problem.

Uniprot ID	Property/Target Protein Name	Origin of dataset	Associated bioactivities	Total number of observations (N-retrieved)	Selected number of observations (N-processed)
-	BBB’s permation	oChem	LogBB	3244	975
P00533	EGFR	ChEMBL	IC_{50}	8479	7134
P42338	PI3Kp110β	ChEMBL	IC_{50}	2416	1870

Data was searched until Dez 2019.

Supplementary Table S2. Description of crystallographic structures selected from PDB for each protein target.

Target	PDB code	Resolution X-ray diffraction	Length	Non-water Atoms	Theoretical Weight	Source Organism	Structure domain	Chain
**EGFR**	1xkk	2.4Å	352 aa	2299	40.32 KDa	Homo sapiens	Kinase catalytic subunit	A
	3w2s	1.9Å	330 aa	2544	37.56 KDa			
	3poz	1.5Å	327 aa	2410	37.3 KDa			
	5u8l	1.6Å	329 aa	2410	37.58 kDa			
**PI3K**	2y3a	3.3Å	1092 aa	9848	125.24 KDa	Mus musculus	Catalytic subunit p110β	A
	4bfr	2.8Å	952 aa	13697	109 kDa			

Data was accessed until March 2020 in **http://www.rcsb.org**.
